# Reversion-inducing cysteine-rich protein with Kazal motifs gene expression and its clinical significance in peripheral T-cell lymphoma

**DOI:** 10.3892/ol.2013.1306

**Published:** 2013-04-16

**Authors:** XIA MAO, LONGLONG LIU, BING ZHANG, DONGHUA ZHANG

**Affiliations:** Department of Hematology, Tongji Hospital, Tongji Medical College, Huazhong University of Science and Technology, Wuhan, Hubei 430030, P.R. China

**Keywords:** reversion-inducing cysteine-rich protein with Kazal motifs, prognosis, peripheral T-cell lymphoma

## Abstract

The reversion-inducing cysteine-rich protein with Kazal motifs (*RECK*) gene was originally identified as a transformation suppressor gene that is widely expressed in normal tissues. In tumor tissues, *RECK* expression levels are significantly reduced, and the downregulation of *RECK* has been implicated in tumors that are more aggressive with a poor prognosis. In the present study, *RECK* expression in peripheral T-cell lymphoma (PTCL; n=82) was examined using immunohistochemistry, and its correlation with clinicopathological factors was analyzed. According to the proportion of positively-stained cells and the staining intensity (SI), the patients were categorized into *RECK*-negative or *RECK*-positive groups. *RECK* expression was observed in 30 of the 82 patients (36.6%). The 3-year survival rate of the patients with *RECK*-positive tumors (65.5%) was significantly high compared with that of the patients with *RECK*-negative tumors (20.3%; P=0.046). Reduced *RECK* expression was found to be significantly correlated with extranodal lymphomatous involvement (P=0.012). The survival analysis showed that *RECK*-negative expression was an independent and significant factor for predicting a poor prognosis. *RECK* status is a useful prognostic factor for assessing the biological behavior in PTCL.

## Introduction

The term peripheral T-cell lymphoma (PTCL) does not refer to the site of involvement, but to the immunophenotype of these tumors that derive from post-thymic (or mature) T cells at various steps of differentiation (1,2). Since natural killer (NK) cells are closely associated with T cells, and share certain immunophenotypic and functional properties, mature T-cell and NK-cell lymphomas are usually classed together (1,2). PTCL comprises of a heterogeneous group of hematological tumors that the World Health Organization (WHO) classification subdivides into specified and unspecified (U) (3). These tumors constitute ∼12% of all lymphoid neoplasms (4,5) and their incidence varies between countries and races, being higher in Japan and other Eastern areas for epidemiological reasons, including the presence of human T-lymphotropic virus 1 (HTLV-1) infections (6–8). These neoplasms often present at an advanced stage in middle-aged/elderly patients at diagnosis (9–11) and most commonly have an aggressive clinical course. Patients succumb rapidly despite prompt therapies. Relapse is common and the prognosis is poor. The rarity of these tumors means that additional studies are required to improve our understanding of their biology.

The reversion-inducing cysteine-rich protein with Kazal motifs (*RECK*) gene was originally isolated using cDNA expression cloning designed to locate transformation suppressor genes against activated ras oncogenes (12). The *RECK* gene is widely expressed in numerous normal tissues and non-neoplastic cell lines, but its expression is low or undetectable in oncogene-transformed fibroblasts or tumor-derived cell lines (13,14). The *RECK* gene encodes a membrane-anchored glycoprotein that is a negative regulator of the matrix metalloproteinases (MMPs), including MMP-2, MMP-9 and MT1-MMP. The restoration of *RECK* expression in malignant cells reduces pro-matrix MMP-9 secretion and suppresses the ability to invade and metastasize, suggesting a role for *RECK* in the regulation of MMPs and tumor invasiveness (13). Numerous studies have reported that a lower expression of *RECK* is associated with a worse prognosis in a variety of cancers (15–19), but little is known with regard to the significance of *RECK* in PTCL.

Therefore, in the present study, the expression of *RECK* was analyzed in patients with PTCL and these data were compared with the clinical and pathological features to determine whether the expression of *RECK* is a predictor of the clinical behavior of PTCL.

## Materials and methods

### Patients and tumor samples

A total of 82 patients with PTCLs who were diagnosed between 2006 and 2010 at the Department of Pathology, Tongji Hospital (Wuhan, Hubei, China), were included in the present study. Approval for the study was obtained from the Medical Ethics Committee of the Tongji Hospital. The specimens and clinical data were collected subsequent to obtaining informed consent in accordance with the Declaration of Helsinki. Samples were obtained at the initial presentation of the patients, then fixed in formalin and embedded in paraffin. The tumor specimens that were analyzed were from biopsies performed prior to chemotherapy. The paraffin blocks were evaluated again to select a representative area. All cases were reviewed carefully and the pathological diagnosis of PTCL was made according to the WHO criteria for the classification of malignant lymphoma following precise immunohistochemical evaluation (20). The clinical features of age, gender, tumor stage, performance status, serum concentration of lactate dehydrogenase (LDH), extranodal lymphomatous involvement, the presence or absence of B symptoms and the International Prognostic Index (IPI) (21), treatment and follow-up were analyzed. The recorded sites of extranodal lymphomatous involvement included the gastrointestinal tract, liver, spleen, lungs, central nervous system and bone marrow (21).

Among the 82 selected patients, there were 55 males (67.1%) and 27 females (32.9%), with an average age of 42.2 years (range, 7–77 years). The histological subtypes were PTCL-not otherwise specified (PTCL-NOS; 28 cases), angioimmunoblastic T-cell lymphoma (22 cases), extranodal NK/T-cell lymphoma, nasal type (16 cases) and intestinal NK/T-cell lymphoma (16 cases). The tumor stages were classified as localized disease (stage 1 or 2) in 21 patients and advanced disease (stage 3 or 4) in 61 patients. There were 42 patients with extranodal lymphomatous involvement (Table I).

The follow-up duration was defined from the date of the initial presentation to the date of mortality or last follow-up. The median follow-up period was 24.1 months (range, 0–48 months). During the follow-up, 38 patients (46.3%) were continuously disease-free, 40 patients (48.8%) succumbed to the disease and four patients succumbed during chemotherapy.

### Immunohistochemistry

*RECK* expression was determined using the streptavidin-peroxidase immunohistochemistry method. Serial 4-*μ*m sections were cut from formalin-fixed, paraffin-embedded blocks and placed on polylysine-coated slides. The serial sections were deparaffinized in three changes of xylene, rehydrated in descending concentrations of ethanol and washed three times for 5 min each with double-distilled water. Following rehydration, the sections were placed in 0.01 M sodium citrate buffer (pH 6.0) for 10 min at 105°C. Subsequent to being cooled to room temperature for 30 min, the specimens were incubated for 30 min at room temperature in 0.3% hydrogen peroxide in methanol to inactivate endogenous peroxidase activity. The sections were then incubated for 30 min at 37°C with phosphate-buffered saline (PBS; pH 7.4) containing 5% bovine serum albumin (BSA; Merck, Darmstadt, Germany), followed by overnight incubation at 4°C with anti-*RECK* mouse monoclonal antibody (Clone 28; BD Transduction Laboratories, San Diego, CA, USA) diluted 1:200 in PBS containing 1% BSA. The sections were washed three times for 5 min in PBS with Tween-20 and incubated for 1 h with biotinylated anti-mouse IgG secondary antibodies (Abcam, Cambridge, UK) diluted 1:300 in PBS containing 1% BSA. Subsequent to rinsing, the immune complexes were visualized using the standard avidin-biotin-peroxidase complex (ABC) method and the sections were then counterstained with Mayer’s hematoxylin and mounted. Positive controls guaranteed the persistent quality of the staining procedure. Negative control slides in the absence of primary antibody were included for each staining.

The expression of *RECK* was independently evaluated by two investigators without knowledge of the patients clinicopathological features. The data from the two investigators were averaged. An evaluation of the *RECK* staining reaction was performed in accordance with the immunoreactive score (IRS): IRS = SI (staining intensity) × PP (percentage of positive cells) (22). The proportional scoring categories of the positively-stained cells were as follows: i) 0, no positivity; ii) 1+, ≤10% positive tumor cells; iii) 2+, 11–50% positive tumor cells; iv) 3+, 50–80% positive tumor cells; and v) 4+, >80% positive tumor cells. The intensity of the *RECK* immunostaining was scored as follows: i) no staining, 0; ii) weak, 1+; iii) moderate, 2+; and iv) intense, 3+. For tumors that showed heterogeneous staining, the main pattern was taken into account for scoring. The IRS was calculated in ≥10 areas at ×400 magnification and ≥1,000 tumor cells were evaluated for each section. Tumor slices scoring at least three points were defined as positive, otherwise they were defined as negative (22).

### Statistical analysis

The clinicopathological characteristics were compared with the expression levels of *RECK* using the χ^2^ test. The Kaplan-Meier method was used to analyze the post-operative survival rate, and the survival differences were analyzed using the log-rank test on the basis of the status of the *RECK* expression. Any factor affecting the prognosis in a univariate analysis was then estimated in a multivariate analysis using Cox’s proportional hazard model with a forward conditional stepwise procedure to determine whether the factor was acting independently. All calculations were performed using SPSS version 13.0 (SPSS, Inc., Chicago, IL, USA) software and P<0.05 was considered to indicate a statistically significant difference.

## Results

### Correlation of RECK protein expression with clinicopathological patient features

**Figure 1:**
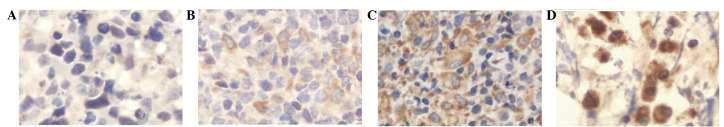
Immunohistochemical detection of reversion-inducing cysteine-rich protein with Kazal motifs (RECK) in peripheral T-cell lymphomas (PTCL). *RECK* immunoreactivity was found in the cytoplasm and plasma membrane of the tumor cells in a granular pattern. (A–D) Examples of lymphomas expressing different levels of *RECK.* (A) No detectable stain (intensity score 0 and proportion score 0). (B) Intensity score 1 and proportion score 1. (C) Intensity score 2 and proportion score 2. (D) Intensity score 3 and proportion score 3. (Original magnification, ×400; Mayer’s hematoxylin stain).

**Figure 2:**
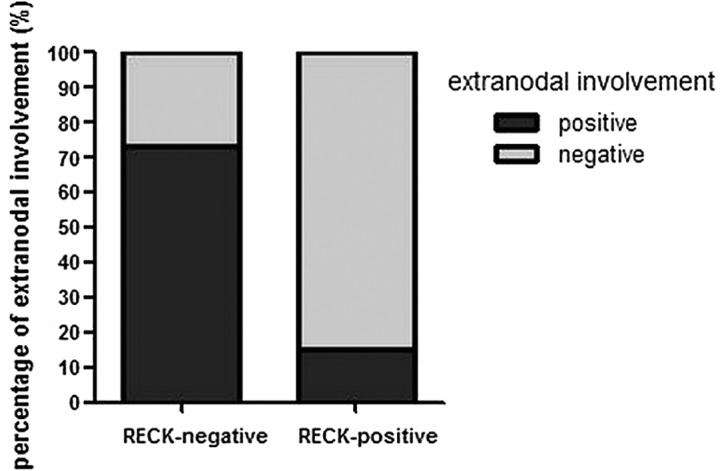
Correlation between reversion-inducing cysteine-rich protein with Kazal motifs (*RECK*) gene expression and extranodal lymphomatous involvement. Positive RECK expression was significantly associated with a lower proportion of extranodal lymphomatous involvement.

*RECK* staining was mainly observed in the cytoplasm and plasma membrane of the tumor cells, often in a granular pattern (Fig. 1). On the basis of multiplying the intensity and proportion scores of *RECK* in the tumors, 30 patients (36.6%) were classified as *RECK*-positive and 52 (63.4%) as *RECK*-negative. No correlation was revealed between the *RECK* status and the age, gender, subtype or tumor stage (Table I). *RECK* expression was inversely correlated with extranodal lymphomatous involvement (P=0.012; Fig. 2).

### Correlation of RECK protein expression with prognostic factors

**Figure 3:**
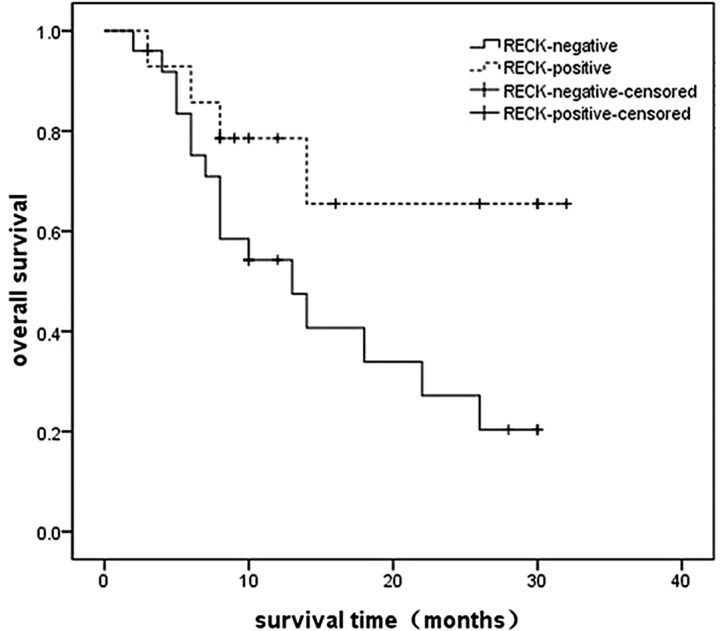
Kaplan-Meier analyses for overall survival for reversion-inducing cysteine-rich protein with Kazal motifs (*RECK*)-positive cases compared with *RECK*-negative cases. The solid line represents negative staining, and the dotted line represents positive staining. The 3-year survival rate of the RECK-positive patients was significantly higher compared with that of the RECK-negative patients.

The univariate analyses of the prognostic factors demonstrated that the *RECK* expression, response to chemotherapy, IPI and tumor stage were significant prognostic factors. No correlations were evident between the prognosis and the age, gender or subtype. The 3-year survival rate of the *RECK*-positive patients was 65.5%, which was significantly higher than that of the *RECK*-negative patients (20.3%; P=0.046; Fig. 3). A multivariate analysis confirmed that *RECK-*positive protein expression was an independent and significant factor for predicting a favorable prognosis.

## Discussion

PTCLs are a biologically diverse and uncommon group of malignant tumors that share an poor prognosis. The cause of the poor survival outcome of patients with PTCL compared with patients with aggressive B-cell lymphomas remains largely unexplained. Possible reasons include the rarity of these disorders and their biological heterogeneity, which make them difficult to study. Moreover, the inclusion of only small proportions of PTCL patients in studies investigating therapies for aggressive B-cell lymphoma has not aided these therapeutic strategies. For the majority of PTCL subtypes, a poor outcome with a 5-year overall survival of ∼30% is reported in the predominance of studies (9,10,23–27). Previous studies have shown that the parameters that may be independent prognostic factors for survival in PTCL, excluding anaplastic large cell lymphoma (ALCL), include age >60 years, disseminated stage, LDH level higher than normal, performance status and a higher IPI and tumor score (10). However, the superiority of these parameters has not been well documented in subsequent studies (28). It may be necessary to investigate the molecular markers predicting the response to chemotherapy, the overall prognosis and the likelihood of extranodal lymphomatous involvement at diagnosis. This may also provide targets for the development of new therapeutic agents.

*RECK*, a novel MMP inhibitor, was first identified by Takahashi *et al* in the NIH3T3 cell line transfected with the v-Ki-*Ras* gene (12). The *RECK* gene encodes a 110 kDa membrane-anchored glycoprotein that contains serine protease inhibitor-like domains and multiple epidermal growth factor-like repeats (12). Initially, *RECK* was believed to be a novel transformation suppressor gene, but it was later identified that *RECK* was able to inhibit the secretion and activity of three MMPs; MMP-2, MMP-9 and MTl-MMP. Numerous oncogenes, including *ras, fos* and *myc,* downregulate the expression of *RECK*, indicating that *RECK* may be a negatively-regulated target of oncogenes (29). A previous study reported that *RECK* is associated with angiogenesis and its appropriate expression inhibits the development of blood vessels (13). Studies have indicated that there is a positive correlation between *RECK* expression in tumors and the survival outcome of patients with other types of tumors, including hepatocellular carcinoma (15), pancreatic cancer (14), breast cancer (16) and non-small cell lung cancer (17). The correlation may therefore be a common feature among numerous tumors and this appears consistent with the previous findings showing that the prognosis for patients that were positive for the expression of *RECK* was better than for those who were negative.

In the present study, *RECK* expression in biopsy specimens and its prognostic significance in patients with PTCL was investigated. It was demonstrated that reduced *RECK* expression was a significant factor for predicting a poor prognosis. The clinicopathological analysis revealed that the expression of *RECK* was not notably correlated with the age, gender, pathological classification or tumor stage of the patients, but that it was associated with extranodal lymphomatous involvement and prognosis in PTCL. The results suggested that positive *RECK* expression was significantly associated with a lower proportion of extranodal lymphomatous involvement and a longer overall survival, although further studies are required in order to confirm this conclusion. In addition, clinicopathological factors were revealed, including *RECK* expression and response to chemotherapy, which may be useful prognostic determinants of favorable overall survival in patients with PTCL.

Numerous studies have suggested that the expression of *RECK* is associated with tumor metastasis and is useful as an informative prognostic indicator for several neoplastic diseases (15–17, 29). In the present study, it was revealed that the patients who were positive for the expression of *RECK* had less extranodal lymphomatous involvement and a longer overall survival; these results were consistent with those of previous reports. The 3-year overall survival rate of this group of patients was 46% and the median survival was 18 months. The 5-year survival rate of patients with PTCLs is reported as ∼30% in the literature (9,10,23–27), which is consistent with the present study.

Certain limitations have been noted in the design of the present study. The study was an initial retrospective analysis of patients with a relatively small sample size, which meant that the study had limited statistical power and ultimately caused the data to generate a certain amount of deviation.

In conclusion, a significant correlation between *RECK* expression in PTCL and extranodal lymphomatous involvement of the patients was identified. A positive correlation between *RECK* expression and the survival rates of the patients was also observed. These data are consistent with the *RECK* model playing an active role in suppressing the malignant phenotypes of PTCL cells. Practically, *RECK* expression may be a good prognostic indicator in PTCL patients, and therapeutic strategies based on *RECK* or its mechanism of action may be of value in the treatment of this disease. Moreover, the clinical use of *RECK* as a prognostic indicator requires further evaluation.

## Figures and Tables

**Table I:** Clinicopathological characteristics for *RECK* expression.

	*RECK* expression		

Variables	Negative	Positive	P-value
Patient Number	52	30	
Age (years)			
>60	12	11	NS
<60	40	19	
Gender			
Male	37	18	NS
Female	15	12	
Histology			
PTCL, unspecified	21	7	NS
Angioimmunoblastic T-cell lymphoma	7	15	
Extranodal NK/T-cell lymphoma, nasal	13	3	
Intestinal NK/T-cell lymphoma	11	5	
Clinical stage			
1, 2	9	12	NS
3, 4	43	18	
Extranodal involvement			
No	14	26	0.012^a^
Yes	38	4	
B symptoms			
No	31	19	NS
Yes	21	11	
Performance status			
0, 1	42	18	NS
2–4	10	12	
LDH			
Normal	8	13	NS
Elevated	44	17	
IPI			
0–2	23	8	NS
3–5	29	22	

a P<0.05. NS, not significant (P>0.05); LDH, lactate dehydrogenase; IPI, internal prognostic index; *RECK*, reversion-inducing cysteine-rich protein with Kazal motifs; NK, natural killer; PTCL, peripheral T-cell lymphoma.
